# Phase-Field Investigation of Lithium Electrodeposition at Different Applied Overpotentials and Operating Temperatures

**DOI:** 10.1021/acsami.2c00900

**Published:** 2022-03-28

**Authors:** Joonyeob Jeon, Gil Ho Yoon, Tejs Vegge, Jin Hyun Chang

**Affiliations:** † Department of Energy Conversion and Storage, Technical University of Denmark, DK-2800 Kgs. Lyngby, Denmark; ‡ School of Mechanical Engineering, Hanyang University, 222, Wangsimni-ro, Seongdong-gu, 04763, Seoul, South Korea; § PhaseTree ApS, DK-2300 Copenhagen, Denmark

**Keywords:** lithium, interfaces, anode, dendrites, phase field, batteries

## Abstract

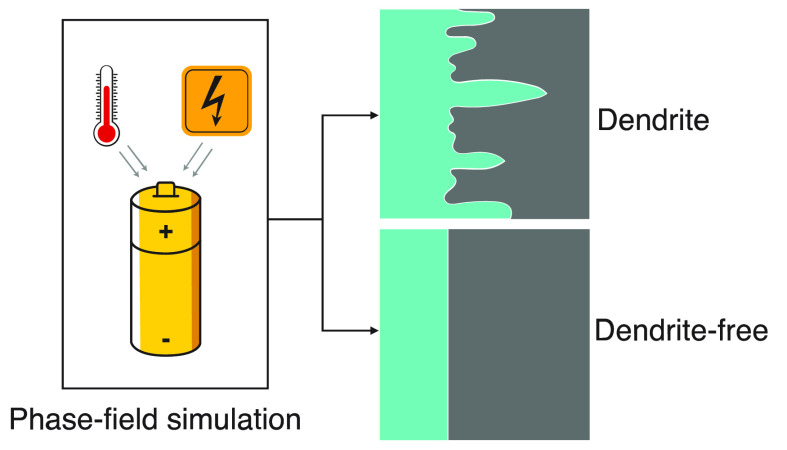

Li metal is an exciting anode for high-energy Li-ion batteries and other future battery technologies due to its high energy density and low redox potential. Despite their high promise, the commercialization of Li-metal-based batteries has been hampered due to the formation of dendrites that lead to mechanical instability, energy loss, and eventual internal short circuits. In recent years, the mechanism of dendrite formation and the strategies to suppress their growth have been studied intensely. However, the effect of applied overpotential and operating temperature on dendrite formation and their growth rate remains to be fully understood. Here, we elucidate the correlation between the applied overpotential and operating temperature to the dendrite height and tortuosity of the Li-metal surface during electrodeposition using phase-field model simulations. We identify an optimal operating temperature of a half-cell consisting of a Li metal anode and 1 M LiPF_6_ in EC/DMC (1/1), which increases gradually as the magnitude of the overpotential increases. The investigation reveals that the temperature dependence identified in the simulations and experiments often disagree because they are primarily conducted under galvanostatic and potentiostatic conditions, respectively. The temperature increase under potentiostatic conditions increases the induced current while it decreases the induced overpotential under galvanostatic conditions. Therefore, the analysis and comparison of temperature-dependent characteristics must be carried out with care.

## 1 Introduction

There is an urgent need for energy storage devices that perform beyond current state of the art Li-ion batteries for various technology sectors, including transportation and grid energy storage. Lithium (Li) metal is considered to be one of the most promising electrode materials for next-generation battery technologies and is used as an anode material for Li-metal,^1−3^ Li–sulfur,^4−6^ and Li–air batteries.^7−10^ Li metal has been investigated in many upcoming battery technologies due to its merits, including a very high energy density of 3680 mA h g^–1^ and a low redox potential of −0.304 V vs the standard hydrogen electrode and a mass density of 0.534 g cm^–3^.^11,12^ These merits make Li a particularly attractive electrode material; the use of Li metal could substantially increase the energy density of batteries.^13^

Despite the advantages, the poor morphological stability of Li metal leads to nonuniform surfaces upon electrodeposition and dissolution during cycling, which has hindered the commercialization of battery technologies based on Li metal.^14,15^ Two nonuniform growth modes of Li are experimentally observed: mossy growth consisting of nanosized whiskers and the fractal growth of dendrites on a microscale when the current density exceeds the critical current density.^15,16^ Both growth modes are detrimental to the battery performance, as whiskers can detach from the current collector to form so-called dead lithium that leads to an irreversible capacity fade, while dendrites can create an internal short circuit that leads to a catastrophic failure.^17^ Some of the recent efforts to inhibit the growth of dendrites include the use of hybrid electrolytes^18,19^ and the application of a protective layer,^20^ which provides mechanical suppression and homogenizes the Li-ion distribution.

It is by no means straightforward to accurately predict the morphology and evolution of dendrites as they are nonlinear processes that depend on many factors such as impurity/defect concentration, applied potential/current, pressure, and operating temperature, to list a few.^12,21−23^ A number of experimental and computational studies have been carried out to understand the underlying mechanisms and correlations between the growth rate and battery operating conditions.^21,22,24−26^ In particular, the growth of dendrites under different applied current densities (galvanostatic)^27,28^ and electric potentials (potentiostatic)^22^ across the interface has been investigated. It was experimentally observed in several reports that increasing the applied current or potential promotes the growth of dendrites when the other conditions are unchanged.^11,26,29,30^ The temperature dependence is often overlooked in the analyses, although it is a critical factor to consider. Commercial batteries are expected to have a wide operating temperature range, and thus, the correlation between the dendrite evolution and the operating temperature needs to be established to ensure the safe and effective operation of batteries. A recent study based on *in situ* optical microscopy and *ex situ* scanning electron microscopy (SEM) revealed a dramatic change in the dendrite growth;^26^ under fixed current density conditions, elevating the operating temperature was observed to result in a larger Li nuclei size and a lower nucleation density. The elevated temperature from internal heating is reported to smooth the surface due to extensive surface migration.^31^

The phase-field method^32,33^ is one of the most popular computational methods for modeling dendrite growth, as it is well-suited to simulate the evolution of the interface between two dissimilar materials. Li dendrite growth is a nonlinear process that depends on various factors such as electrochemical reactions, applied overpotential, operating temperature, and Li-ion concentration in the electrolyte.^28,34^ Consequently, nonlinear phase-field models that directly include the contributions of these factors have been used to predict the dendrite evolution.^23−25,35^ The effect of the applied overpotential across the interface on the dendrite evolution has been studied in the past,^23,24^ as it is an externally controllable factor that directly affects the dendrite growth mechanism and rate. Hong and Viswanathan^23^ investigated the evolution of the Li electrode when three different overpotentials (i.e., −0.32, −0.45, and −0.50 V) were applied across the electrode–electrolyte interface. They found that the Li ions get consumed at a higher rate through electrochemical reactions under a higher overpotential. The electrodeposition process is more prone to become transport-limited as the Li ions at the interface become depleted. The presence of small peaks due to the inhomogeneity boosts the ion transport in its vicinity due to the migration from the surrounding valley regions, thereby increasing the electrochemical reaction rate near the peak while further depleting the Li-ion concentration in valleys.^23^ Interestingly, Hong and Viswanathan^23^ argued that the key feature that distinguishes the low-overpotential regime (no dendrite formation) from the high-overpotential regime (dendrite formation) is the concentration of Li ions at the interface relative to that of the bulk; the Li concentration at the interface is higher than that of the bulk value when the overpotential is low, while the opposite is true when the overpotential is high.

Only a limited number of computational studies have investigated the thermal effect on the dendrite evolution. Recent works^21,22^ include derivation of a temperature field using a heat transfer model and coupling it with diffusion coefficients. This elegant approach is capable of not only including the change in the ambient temperature but also the internal heat generation, convection and radiation. A simpler model that substitutes different temperature values in the governing equations without any coupling term has also been used to investigate the electrodeposition of zinc.^36^ Gao and Guo^37^ accounted for heat generation and diffusion in their temperature field to investigate the internal temperature distribution. However, the state of the art phase-field models only account for the local temperature effect on diffusivity values, and a comprehensive inclusion of the thermal effect on other physicochemical parameters remains missing.

This work aims to fully integrate the thermal effects on various aspects of the nonlinear phase-field model to assess the contribution of the operating temperature and applied overpotential to the dendrite formation and growth rate. We included the temperature dependence of the electrode conductivity, electrolyte conductivity, surface tension, exchange current density, and the Li-ion diffusivity in the electrolyte. The electrodeposition process is simulated at varying operating temperatures and applied overpotentials in order to assess the contribution of the two, and the resulting morphology evolution is analyzed in terms of the tortuosity and the maximum height of the dendrite when the same amounts of Li ions are deposited onto the electrode. Our results show that the induced current across the interface increases when the temperature is elevated, and a direct comparison of the temperature dependence based on galvanostatic and potentiostatic results should be avoided. A comparison of surface modulation after depositing the same amount of Li at different temperatures and applied overpotentials revealed that the dendrite growth rate as a function of the amount of deposited Li is similar across cases. A critical factor to consider is the onset point at which dendrites start to form, which is determined via setting threshold criteria for the dendrite height and tortuosity. Using the developed phase-field model, we have identified that increasing the magnitude of the overpotential lowers the onset point and determined an optimal operating temperature under different values of applied overpotential.

It is worth noting that the influence of the solid–electrolyte interphase (SEI) is often omitted in the phase-field model or included in a greatly simplified manner. The SEI is reported to influence the formation of dendrites^38^ and the growth of whiskers and mossy structures.^39^ However, the exact mechanism behind the formation and growth of SEI is not fully understood,^40^ making it difficult to incorporate it in the phase-field model for simulating dendrite growth. While some phase-field models are used to simulate the formation and evolution of the SEI layer,^41,42^ the phase-field models developed for simulating the dendrite evolution often neglect the SEI effect or include it on an ad hoc basis in the form of a modification factor for the current density^35^ or a noise field to the interface.^43^ The influence of the SEI layer is omitted in the present model, as the focus was on determining the onset point of dendrite formation at different operating temperatures and applied overpotential. However, it is desirable to incorporate the formation and evolution of the SEI in future models to investigate its effect on surface modulation during electrodeposition.

## 2 Methodology

### 2.1 Model Overview

**Figure 1:**
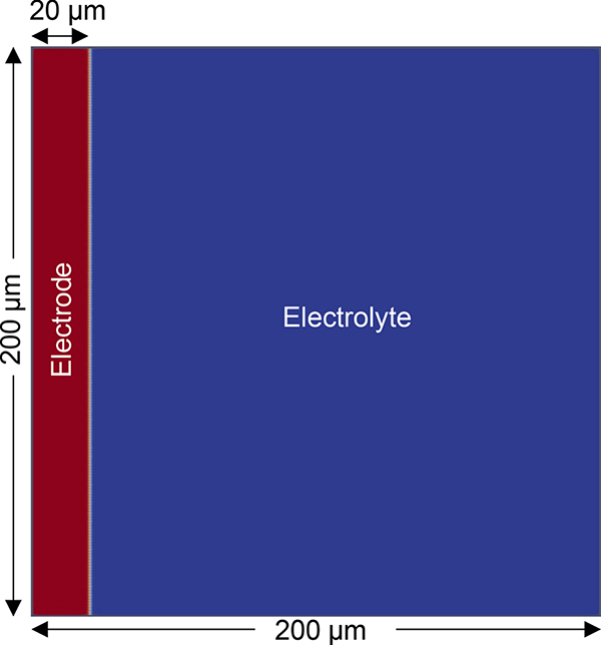
Initial geometry of the simulation cell before electrodeposition.

We investigated a half-cell system consisting of an Li metal anode and 1 M LiPF_6_ in EC/DMC (1/1). It is noted that the same simulation method can be applied to systems consisting of different electrolytes and other metal electrodes such as sodium and potassium metals, given that the simulation parameters are modified accordingly. The two-dimensional simulation cell has dimensions of 200 μm × 200 μm; the anode has an initial thickness of 20 μm, while the remaining 180 μm is occupied by the electrolyte, as shown in Figure 1. The Li^+^ ion from the electrolyte approaches the Li-metal surface during the electrodeposition process, reacting with the electrons in the electrode and becoming reduced to Li atoms. The electrodeposition process can be described using a simple chemical reaction:
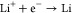
1

The two phases of the system—Li metal anode and electrolyte—are distinguished using the order parameter, ξ. The order parameter is a continuous parameter that represents the phase of the system, and it ranges from 0 to 1. ξ = 0 corresponds to the electrolyte phase, while ξ = 1 corresponds to the Li metal phase. The interface between the electrode and electrolyte has a finite thickness, where the value of ξ lies between 0 and 1. All of the reported results are generated using the phase-field simulation module of PhaseTree.^44^

### 2.2 Phase-Field Model

A description of the free energy of the interface is important for constructing a phase-field model. The interfacial free energy of the Ginzburg–Landau type^32^ is expressed using the multiwell potential and gradient coefficient energy,^24,25,34,35,37^ which makes a sharp interface energy with smooth profiles of the phase fields. The system considered for this work is represented using a double-well potential with two equilibrium states, ξ = 0 and ξ = 1. The interfacial free energy, *U*, is written as
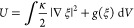
2where  and *g*(ξ) are the terms describing the gradient energy density and double-well potential, respectively. The gradient energy term models the diffusion process that smooths out the order parameter, while the double-well potential term counteracts such smoothing by separating the values through the potential barrier.^32^ κ is a gradient coefficient defined as , where γ is the surface tension and δ is the interface thickness. The double-well potential is written as *g*(ξ) = ωξ^2^(1 – ξ^2^), where the barrier height, ω, is defined as .^24,25,32^

The temporal evolution of the order parameter is related to the interfacial free energy by
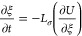
3where *L*_σ_ is the interface mobility. Substituting eq 2 into *U* in eq 3 leads to a well-known Allen–Cahn equation:
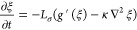
4The Allen–Cahn equation is the first component describing the temporal evolution of the electrode–electrolyte interface during electrodeposition.

### 2.3 Modified Butler–Volmer Equation

The description of the temporal evolution thus far does not include the change in the energy due to the electrochemical reactions. The electrochemical reaction kinetics is described using a modified Butler–Volmer equation, which can be written as
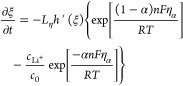
5 is the electrochemical reaction kinetic coefficient, where *V*_m_ is the molar volume of Li and *i*_0_ is the exchange current density.  is the interpolating function that is generally used in phase-field modeling, and the most commonly used function is *h*(ξ) = ξ^3^(6ξ^2^ – 15ξ + 10), which is also used in this work. While it satisfies the condition of smoothly interpolating ξ from 0 to 1,^45^ the function also has its derivative, *h*′(ξ) = 30ξ^2^(1 – ξ)^2^, that limits the electrochemical reactions to take place only at the interface as *h*′ becomes zero when the value of ξ approaches 0 or 1. *F* is Faraday’s constant (96485 C mol^–1^), *R* is the gas constant (8.314 J mol^–1^ K^–1^), α is the charge transfer coefficient (set to 0.5 in this work), *n* is the number of electrons transferred in the reaction (1 for Li electrodeposition as described in eq 1), and *T* is the temperature in kelvin. The activation overpotential, η_α_, is defined as η_α_ = ϕ – *E*_0_, where ϕ is the applied overpotential and *E*_0_ is the standard equilibrium half-cell potential, which is set to zero. *c*_0_ and  are respectively the initial and local Li-ion molar ratios of the electrolyte, where the initial molar ratio corresponds to the molar ratio of the bulk electrolyte that serves as the baseline for assessing the local molar ratio near the interface.

The overall temporal evolution of the order parameter (i.e., the evolution of surface morphology) can be described via the superposition of eqs 4 and 5,^23−25,34^ which is expressed as
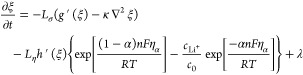
6Note that an additional term that represents Langevin noise, λ, is added to eq 6 to account for the perturbation in the system due to surface defects and thermal variations that may trigger the formation of the dendrite nucleus. The magnitude of Langevin noise was set to 0.04 for this work.

### 2.4 Modified Diffusion Equation

The temporal evolution of chemical potential, μ, derived from the mass conservation law is written as^23,25^
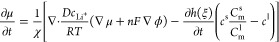
7where the susceptibility factor, χ, is defined as
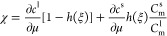
8*C*_m_^l^ and *C*_m_^s^ are site densities of the liquid (electrolyte) and solid (electrode) phases, respectively. Similarly, *c*^l^ and *c*^s^ respectively represent the molar ratios of solid and liquid phases and are related to the chemical potential of the system by
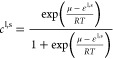
9Here, ε^l,s^ is the difference in the chemical potential of Li species with respect to that of the neutral components at the initial equilibrium. The local Li-ion molar ratio of the electrolyte is related to the molar ratio of the liquid phase via .

### 2.5 Charge Conservation Equation

The system is electrically neutral, and its charge conservation is described using Poisson’s equation, which is written as
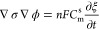
10where σ is the effective conductivity and is related to the conductivity of the electrode, σ^s^, and electrolyte, σ^l^, using the interpolation function as
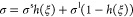
11

### 2.6 Temperature Dependence of the Parameters

**Figure 2:**
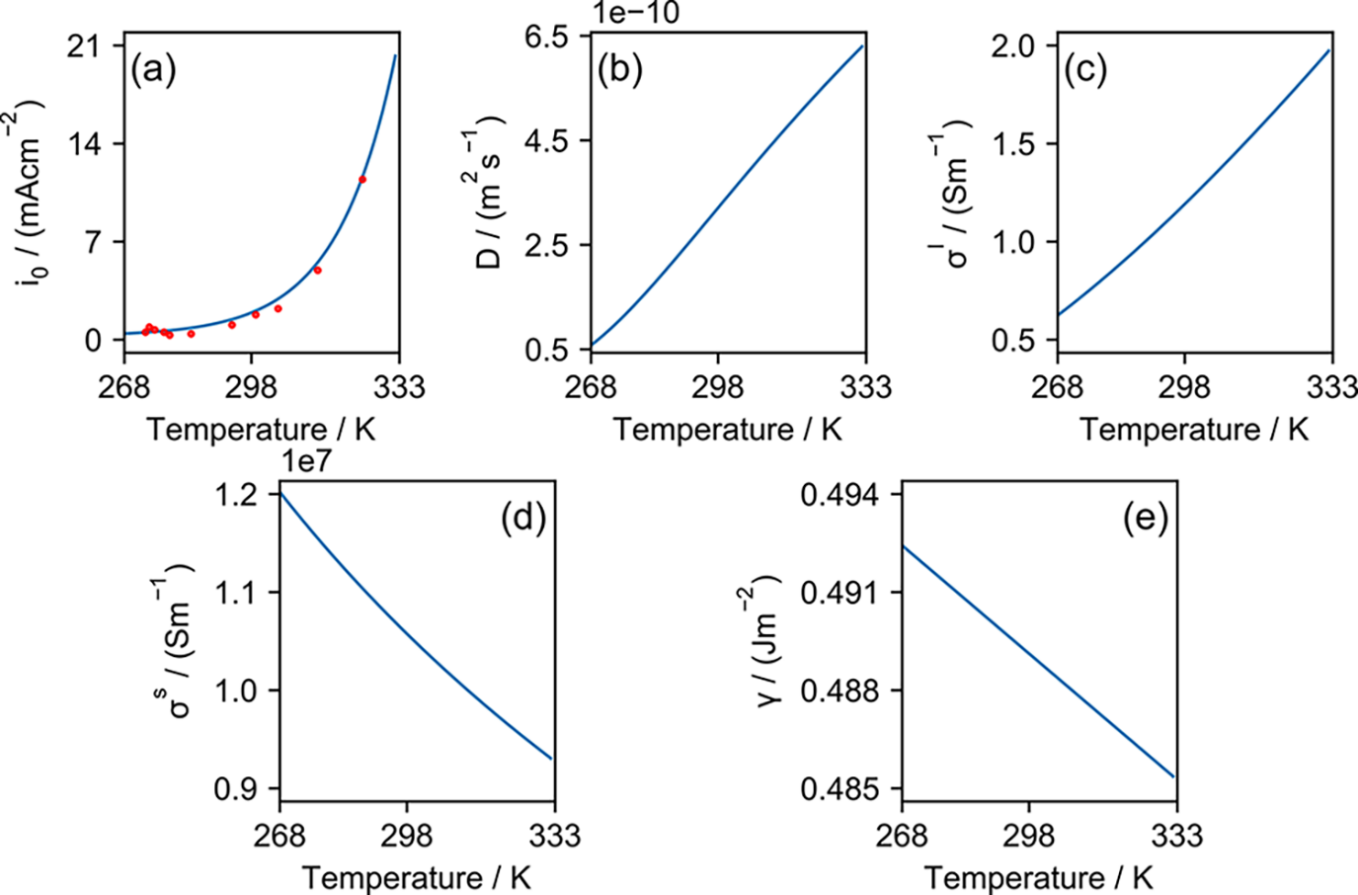
Temperature-dependent values of (a) exchange current density, (b) ionic diffusivity of the electrolyte, (c) conductivity of the electrolyte, (d) conductivity of the electrode, and (e) surface tension of the electrode for the temperature ranging from 268 to 333 K.

The description of the phase-field model thus far provides an overview without the influence of the temperature of the system. The effect of the temperature is directly included with the temperature term, *T*, in cases such as the modified Butler–Volmer expression in eq 6. However, most of its effect is reflected through the temperature dependence of the physicochemical parameters. The temperature dependence of the parameters—exchange current density, ionic diffusivity, electrode and electrolyte conductivity, and surface tension—are shown in Figure 2.

The exchange current density, *i*_0_, across the Li metal anode and 1 M LiPF_6_ in EC/DMC (1/1) is taken from the work of Hess,^46^ who reported the experimentally measured values at temperatures between 253.55 and 344.35 K. The reported values are shown as red dots in Figure 2a). The experimental data were fitted using an exponential regression model (shown as a blue line). The fitted exchange current in mA cm^–2^ is expressed as
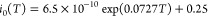
12

The temperature dependence of the ionic diffusivity was obtained from the experimental measurements of Valøen and Reimers,^47^ who reported that the ionic diffusivity, *D*, in m^2^ s^–1^ is
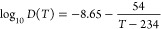
13for *T* between 263 and 333 K.

The conductivity values are also extracted from the experimentally obtained values at varying temperatures. The electrolyte conductivity, σ^l^, of 1 M LiPF_6_ in EC/DMC (1/1) in S m^–1^ is expressed as^47^
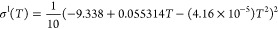
14for temperatures between 263 and 333 K. The resistivity, *R*, of Li metal in Ω m is related to the temperature as
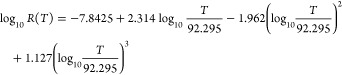
15for temperatures ranging from 92.295 to 453.6 K according to the report by Chi.^48^ The conductivity of the Li metal electrode, σ^s^, in S m^–1^ is the reciprocal of the resistivity:
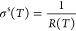
16Another parameter that is known to have temperature dependence is the surface tension of Li metal, γ. Its value is reported^49^ to be
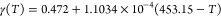
17for temperatures between 0 and 453.15 K, where its value is in J m^–2^. The rest of the parameters that do not depend on temperature are summarized in Table 1.

**Table 1:** Constant Parameters of the Phase-Field Model and Their Normalized Values

symbol	name	value	normalized value	ref
*L*_σ_	interfacial mobility	2.5 × 10^–6^ m^3^ J^–1^ s^–1^	6.25	(24)
*n*	no. electrons transferred	1	1	
δ	interface thickness	1 μm	1	(23)
α	transfer coefficient	0.5	0.5	(50)
*C*_m_^s^	site density of electrode	7.64 × 10^4^ mol m^–3^	76.4	(21, 51)
*C*_m_^l^	site density of electrolyte	1.44 × 10^4^ mol m^–3^	14.4	(23)
*c*^0l^	initial Li electrolyte molar ratio	0.067159	0.067159	(23)
*c*^0s^	initial Li electrode molar ratio	0.999999	0.999999	estimated

Finally, the chemical potential difference between the Li and neutral species at the initial equilibrium, ε^l,s^, is estimated using the initial molar ratio of Li species, *c*^0l,0s^. The approximation is written as  on the basis of the work of Cogswell.^25^ Since the initial molar ratios *c*^0l^ and *c*^0s^ are 0.067159 and 0.999999, respectively, ε^l^ = 2.631*RT* and ε^s^ = −13.8*RT*.

### 2.7 Numerical Settings

The phase-field model solves the temporal and spatial evolution of three parameters: the order parameter, the chemical potential, and the electric overpotential. Each of these parameters needs initial and boundary conditions defined in addition to the governing equations described through eqs 6, 7, and 10. The boundary conditions of the three parameters are shown in Table 2.

**Table 2:** Boundary Conditions Used for the Phase-Field Model

param	boundary (μm)	boundary condition type	value
ξ	*x* = 0	Dirichlet	1
	*x* = 200	Dirichlet	0
	*y* = 0	Neumann	0
	*y* = 200	Neumann	0
			
μ	*x* = 0	Dirichlet	0
	*x* = 200	Dirichlet	0
	*y* = 0	Neumann	0
	*y* = 200	Neumann	0
			
ϕ	*x* = 0	Dirichlet	ϕ_applied_^a^
	*x* = 200	Dirichlet	0
	*y* = 0	Neumann	0
	*y* = 200	Neumann	0

a The value of the overpotential applied across the interface.

The initial geometry shown in Figure 1 is represented via the distribution of order parameter at *t* = 0 s
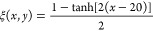
18which represents the electrode thickness of 20 μm with a smooth transition from the electrode to the electrolyte at the interface. The initial distribution of electric potential is set as
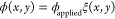
19such that the electrode and electrolyte has the potential of ϕ_applied_ and 0 in the beginning of the simulation. The chemical potential was initially set to zero across the entire domain. The 200 μm × 200 μm domain is represented with a 200 × 200 crossed mesh, and it is solved using Newton’s iterative method with adaptive time steps. The distribution of order parameter, chemical potential, and electric overpotential are saved at 1 s time intervals. The normalization factors of length, time, temperature, moles, energy, and conductance are 1 μm, 1 s, 1 K, 1 × 10^–15^ mol, 2.5 × 10^–12^ J, and 1 × 10^–6^ S, respectively.

### 2.8 Assessment of the Surface Modulation

The morphology of dendrites can vary widely depending on many factors such as types of electrolytes used, temperature, pressure, and current density.^52^ As such, a quantitative assessment scheme is needed to evaluate the surface modulation to determine the presence of dendrites and the extent to which they have grown. In this work, we are using the height of the dendrite and the tortuosity of the surface to quantitatively describe the surface modulation. The dendrite height is defined as the difference between the average height of the Li metal and the maximum height of its peaks; it is a measure of the peak height with respect to that of the average. The tortuosity of the surface, on the other hand, describes the “roughness” of the surface upon electrodeposition and is defined as the ratio between the length of the curved path and the straight path connecting the two end points. In other words, tortuosity is defined as , where *l*_c_ and *l*_s_ correspond to the length of curved and straight paths, respectively. The length of the straight path, *l*_s_, is the length of the straight line connecting the electrode surface at cell boundaries at *y* = 0 μm and *y* = 200 μm. On the other hand, the length of the curved path, *l*_c_, is taken by measuring the length of the path that traces the electrode surface from *y* = 0 μm to *y* = 200 μm. A perfectly smooth surface will have τ = 1, and the roughness of the surface will increase the tortuosity.

**Figure 3:**
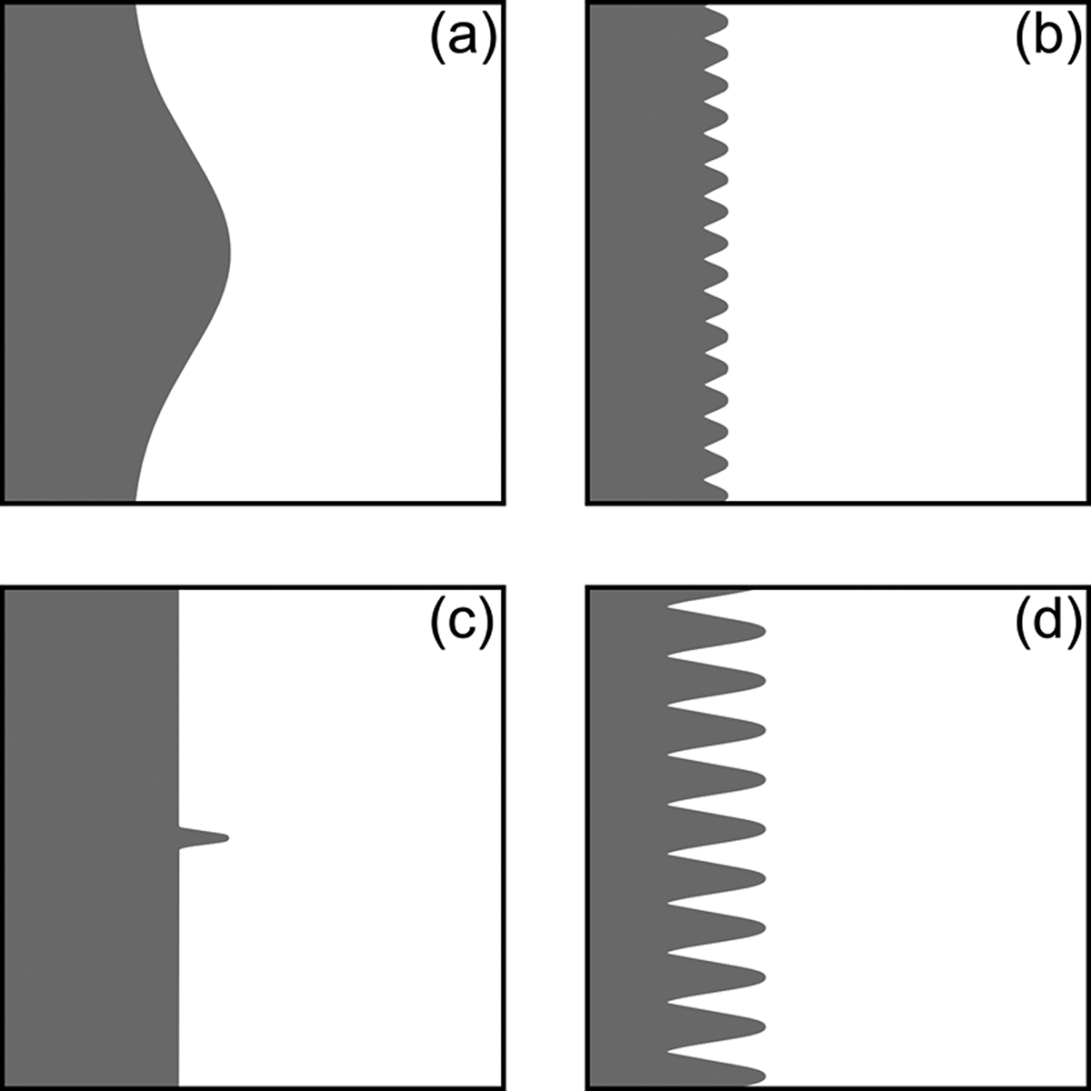
Schematics of surfaces with different dendrite heights and tortuosities: (a) height 20 μm, tortuosity 1.08; (b) height 5 μm, tortuosity 1.94; (c) height 20 μm, tortuosity 1.16; (d) height 20 μm, tortuosity 4.26.

The effects of dendrite height and tortuosity are illustrated in Figure 3. It is seen from Figure 3a that assessing the surface morphology on the basis of the dendrite height alone can be misleading. A hill-like shape with a gradual change in height leads to a low tortuosity value, and such a morphology does not represent a scenario of dendrite formation. In contrast, a rough surface free of any noticeable dendrites has a high tortuosity with low dendrite height, as shown in Figure 3b). The first two cases demonstrate that neither the height nor the tortuosity should be used alone to quantitatively evaluate the surface morphology. The schematic shown in Figure 3c has a needlelike extrusion on an otherwise perfectly smooth surface. The tortuosity is noticeably higher than that of the hill-like pattern even in the presence of a single needlelike dendrite, and the tortuosity becomes significantly higher in the presence of multiple dendrites, as shown in Figure 3d. Therefore, we use both the dendrite height and tortuosity to assess the presence of the dendrite: i.e., both the dendrite height and tortuosity should exceed their threshold values for the surface to be classified to have dendrites. It is noted that the values of dendrite height and tortuosity in Figure 3 are chosen arbitrarily to demonstrate the need of using both height and tortuosity in assessing the surface modulation, and these values should not be interpreted meticulously.

## 3 Results and Discussion

The temperature and overpotential are two control parameters considered in this study. The rest of the system parameters are kept constant, except for their dependence on the temperature. The considered temperature range is from 268 to 333 K with a 5 K increment. The temperature range was selected on the basis of the availability of experimental reports that provide the necessary physicochemical parameters for the phase-field model; a temperature range is selected such that all temperature-dependent parameter values are available. The range of applied overpotential was determined on the basis of the work of Hong and Viswanathan,^23^ which determined that dendrites form at −0.45  and −0.50 V but not at −0.32 V at room temperature. On the basis of their report, this work investigates the effect of overpotential by considering the range between −0.30  and −0.44 V with a 0.02 V increment.

### 3.1 Predicting Dendrite Formation Using the Li-Ion Concentration Profile

**Figure 4:**
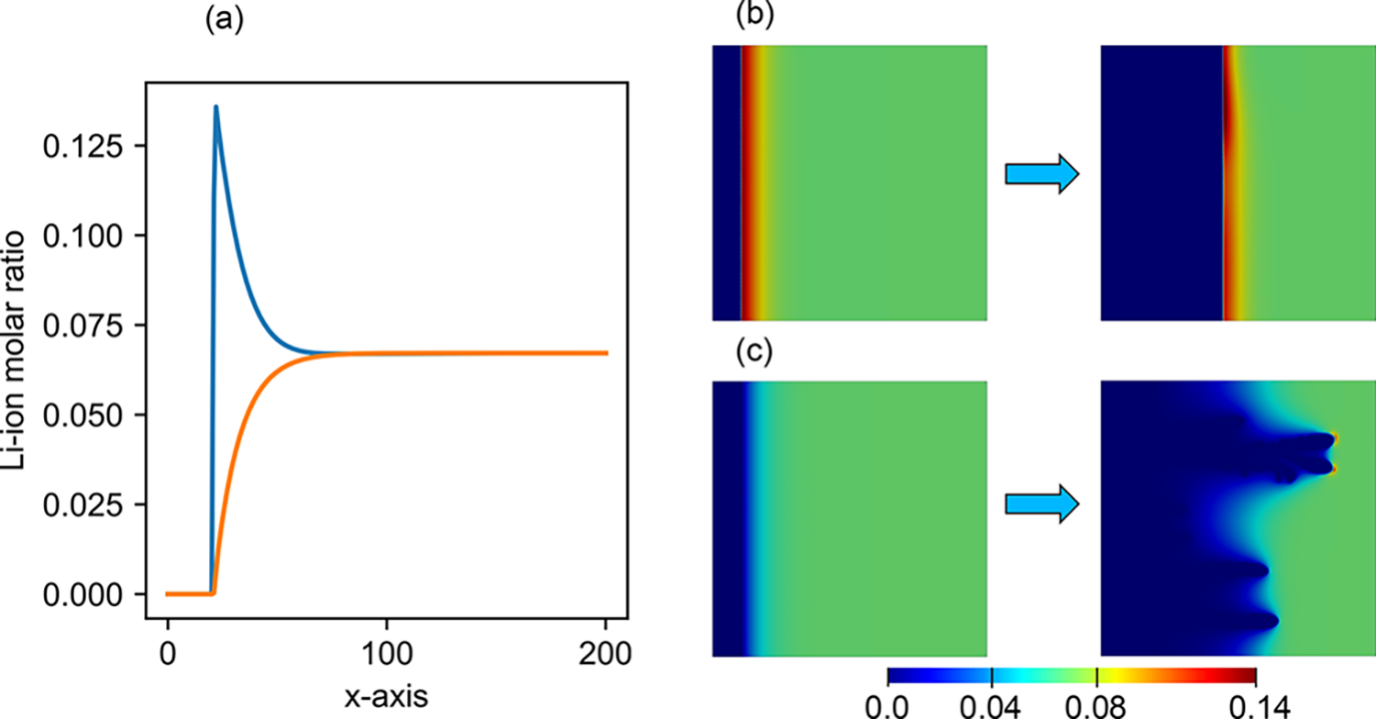
Correlation between the Li-ion concentration profile and the formation of dendrites. (a) The Li-ion concentration at the interface can be higher (blue, −0.30 V applied at 268 K) or lower (orange, −0.44 V applied at 333 K) than that of the electrolyte bulk. (b) Dendrites do not form when the interfacial Li-ion concentration is higher than that of the bulk. (c) Dendrite form when the interfacial Li-ion concentration is lower than that of the bulk.

One crucial finding of Hong and Viswanathan^23^ was that dendrite formation could be predicted early in the electrodeposition simulation based on the Li-ion concentration profile. In particular, it was observed that dendrites form when the Li-ion concentration at the interface falls below that of the bulk. For the cases where no surface modulation is observed, on the other hand, the Li-ion concentration at the interface was mostly higher than that of the bulk during electrodeposition, although it oscillates above and below the bulk value. The difference was understood as being a result of direct competition between the ionic transport and electrochemical reaction.^23,53^ The surface grows uniformly when the process is reaction-limited (i.e., the reaction rate is slower than the transport rate), and the interface has an accumulation of Li ions. In contrast, a transport-limited process leads to a depletion of ions at the interface that causes an inhomogeneity of electrochemical reactions at the surface, which in turn promotes the dendrite formation. The two scenarios are illustrated in Figure 4. We investigated the generalizability of their observation as the first step, since the original investigation was limited to room temperature (300 K). More specifically, we demonstrate that the Li-ion concentration profiles early in the simulation can be used to predict the dendrite formation later in the electrodeposition process, as depicted in Figure 4 for different applied overpotentials and operating temperatures.

**Figure 5:**
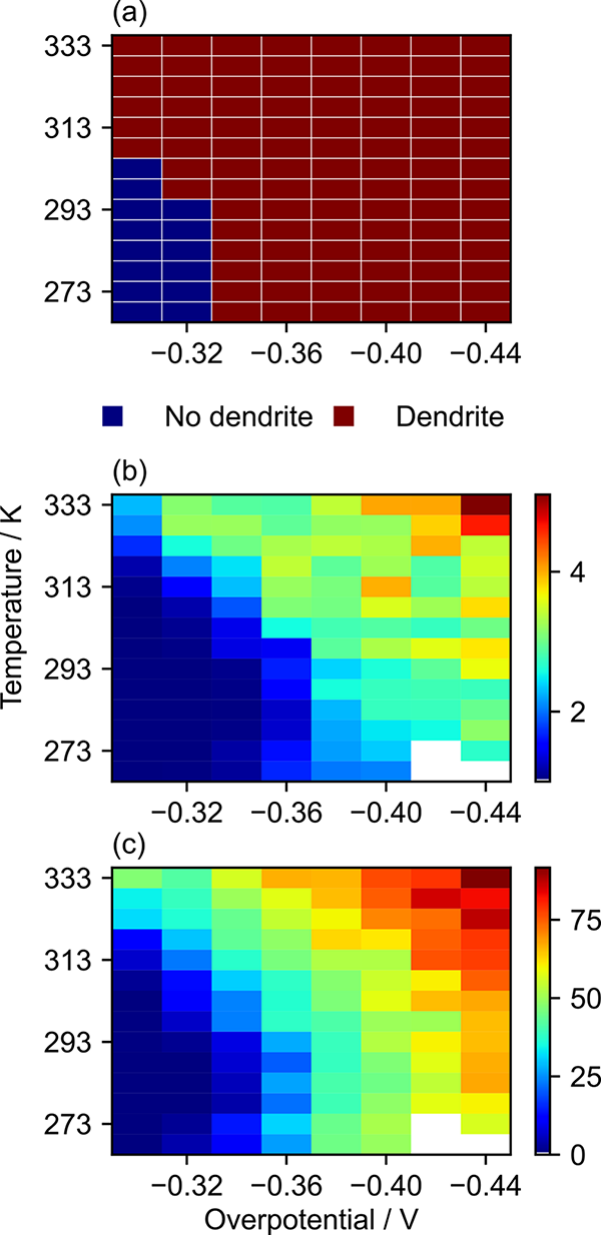
Surface modulation of the Li metal upon electrodeposition at different overpotentials and temperatures. (a) Prediction of the dendrite formation based on the Li-ion concentration profile early in the electrodeposition process. Tortuosity (b) and dendrite height (c) when the peak Li height reaches *x* = 150 μm. White regions in (b) and (c) represent the missing data due to the numerical instability introduced due to the low operating temperature and high magnitude of the overpotential.

The formation of dendrites is predicted for all simulated electrodeposition conditions using the Li-ion concentration profile, as shown in Figure 4. The predictions for all cases are shown in Figure 5a, where the formation of dendrites is marked in blue while the lack of dendrites upon electrodeposition is marked in red. The lack of dendrite formation (labeled as “no dendrite” in Figure 5a) corresponds to the case where the Li-ion concentration profile exhibits a pattern similar to the blue curve in Figure 4a, resulting in the deposition process shown in Figure 4b. In contrast, the case where the dendrites form (labeled as “dendrite” in Figure 5a) corresponds to the orange curve in Figure 4a, resulting in the deposition process shown in Figure 4c. The prediction shows a clear pattern where no dendrites are formed when Li electrodeposition takes place at a low operating temperature and applied overpotential, while the opposite is true for a high temperature and overpotential. The fact that a high applied overpotential promotes the dendrite formation agrees well with previous simulations^23,24,28,34^ and experimental^11,30^ observations. It is noted that the absence of dendrites at 298 K under a −0.32 V overpotential disagrees with the results reported by Hong and Viswanathan,^23^ although their simulation conditions were very similar (*T* = 300 K and ϕ = −0.32 V). We verified that the discrepancy originates from the difference in the electrochemical reaction kinetic coefficient values used in the model; our temperature-dependent parameter value differs from the value they used, and we confirmed that modifying the coefficient to match their value led to the same results as reported previously. However, we emphasize that it is not advised to meticulously interpret the threshold temperature/overpotential value for dendrite formation, since they can shift somewhat on the basis of the parameter values used.

The tortuosity and dendrite height of the surface have been analyzed to verify the validity of the prediction based on the Li-ion concentration profile. It is important to analyze the surface modulation after a sufficient electrodeposition process to avoid a premature assessment. However, it is difficult to also satisfy the condition of having the same amount of Li ions deposited across all simulation conditions; dendrites start to grow almost immediately when both the temperature and overpotential are high, leading the dendrite to reach an *x* = 200 μm boundary even when a relatively small amount of Li ions is deposited. Consequently, the tortuosity and dendrite height are taken when the peak Li height reaches *x* = 150 μm, and the results are shown in Figure 5b,c. The peak height of 150 μm is chosen to avoid the tip from getting too close to the cell boundary.

It can be seen that the prediction based on the concentration profile in Figure 5a agrees well with the tortuosity and dendrite height trend shown in Figure 5b,c. The tortuosity and dendrite height are both low for a low temperature and overpotential and high for a high temperature and overpotential; a clear pattern can be seen in the color map of Figure 5b,c, where the color shifts from blue to red on traversing from the bottom-left corner to the top-right corner. The transition from no dendrite to dendritic regions represents the shifted balance between reaction-limited and transport-limited electrodeposition processes. The electrodeposition is reaction-limited when the temperature and overpotential are low. The shift from no dendrite to dendritic growth at an increased overpotential and temperature indicates that the electrodeposition gradually becomes a transport-limited process. Although there are some fluctuations, it is observed that the overpotential increase results in an increase in tortuosity and dendrite height. Although the general trend was the same for the temperature, it was noticed that the dendrite height undergoes an initial decrease upon an increase in temperature, hitting the minimum value when the temperature is between 278 and 298 K, which increases again as the temperature is increased further. The gradual change in tortuosity and dendrite height also reveals that there is no sudden “shift” from a dendrite-free to a dendritic regime. Consequently, one can set a heuristic condition for determining the presence of the dendrite on the surface by setting a threshold on tortuosity and dendrite height.

The tortuosity and dendrite height threshold values can be determined if the maximum tortuosity and dendrite height in the dendrite-free regime is lower than those of the dendritic regime. We observed one outlier at *T* = 268 K and ϕ = −0.32 V, where the initial Li-ion concentration profile indicated the absence of dendrites upon electrodeposition. Interestingly, the electrodeposition process switched from reaction-limited to transport-limited in this case, resulting in the dendrite formation at a later stage with the final tortuosity and dendrite height of 1.03 and 3.88 μm, respectively. Except for this outlier, the distribution of the tortuosity and dendrite height in Figure 5b,c agreed well with the boundary between the dendrite-free and dendritic regions shown in Figure 5a. The maximum tortuosity and dendrite height in the dendrite-free region are 1.012 and 1.93 μm, respectively. The minimum tortuosity and dendrite height in the dendritic region are 1.015 and 2.07 μm, respectively, indicating a smooth transition between the dendritic and dendrite-free regions. Therefore, we set a threshold where surfaces with a tortuosity larger than 1.014 and a dendrite height higher than 2.05 μm are considered to have dendrites. These thresholds are used to determine the onset condition where the dendrites start to form on the surface.

### 3.2 Influence of Temperature and Overpotential on Electrodeposition Rate and Surface Modulation

**Figure 6:**
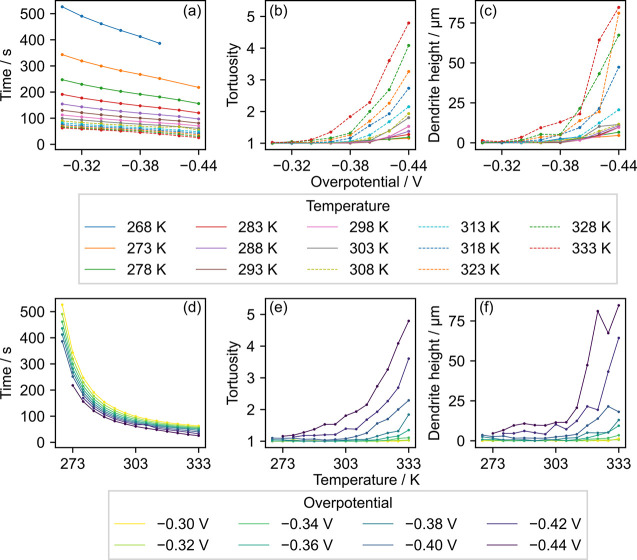
Influence of overpotential and temperature on (a, d) the time it takes to reach the average Li height of 55 μm, (b, e) the tortuosity of the surface, and (c, f) the dendrite height when the average Li height is 55 μm.

The influences of overpotential and temperature on the electrodeposition rate, tortuosity, and dendrite height are compared next. The analysis was carried out using the snapshot taken when the average height of the Li electrode is the same to ensure that a systematic comparison is made when the same amount of Li is deposited. In principle, the height can be chosen arbitrarily without altering the conclusion drawn from the investigation. In practice, taking a snapshot too early (e.g., *x* = 21 μm) does not let the system evolve sufficiently to discern the influence of the temperature and overpotential. On the other hand, taking the snapshot too late leads to scenarios where dendrites reach the simulation cell boundary at *x* = 200 μm for the cases where dendrites start to form almost immediately. Some dendrites already reach the simulation cell boundary when the average height is only 60 μm. Consequently, an average height of 55 μm was selected to ensure that a sufficient electrodeposition process takes place while dendrites do not reach the cell boundary at *x* = 200 μm for all cases. The time it takes to reach the average height of 55 μm, the tortuosity, and the dendrite height as a function of overpotential and temperature are shown in Figure 6.

Figure 6a,d show a clear correlation between the time it takes to reach the average height of 55 μm and both the overpotential and temperature. The elapsed time decreases monotonically as the magnitude of the applied overpotential is increased, indicating that the electrodeposition rate (or electric current), a reciprocal of the elapsed time, increases as the applied overpotential is increased. Therefore, a qualitative comparison can be made between galvanostatic and potentiostatic measurement patterns for varying overpotential/current at a fixed temperature to assess their influence. On the other hand, the elapsed time displays a sharp decay as the temperature is increased (Figure 6d), which highlights an implication in making a direct comparison between the temperature dependence of galvanostatic and potentiostatic electrodeposition. The induced electric current increases as a function of temperature when the overpotential is fixed. In other words, increasing the temperature under potentiostatic conditions will increase the induced current while it decreases the induced overpotential under galvanostatic conditions. The interpretation drawn from our simulations agrees well with the experimental observations by Yan et al.,^26^ where the induced overpotential in the galvanostatic setting is decreased when the temperature is increased. Therefore, it is important to consider both galvanostatic and potentiostatic processes to gain a deeper understanding of the Li nucleation mechanism in future studies.^30^

The tortuosity and dendrite height both show a nonlinear growth as a function of the applied overpotential (Figure 6b,c); the tortuosity and height both increase rapidly after the dendrite starts to form, and the magnitude of the onset potential becomes higher as the temperature is increased. A similar pattern is observed for the temperature dependence (Figure 6e,f), where both the tortuosity and dendrite height grow rapidly after the onset temperature, and the onset point becomes lower as the magnitude of the applied overpotential is increased. It is also found that the tortuosity and dendrite height show a similar temperature- and overpotential dependence, which allows one to estimate the evolution of tortuosity or dendrite height on the basis of the evolution pattern of another.

**Figure 7:**
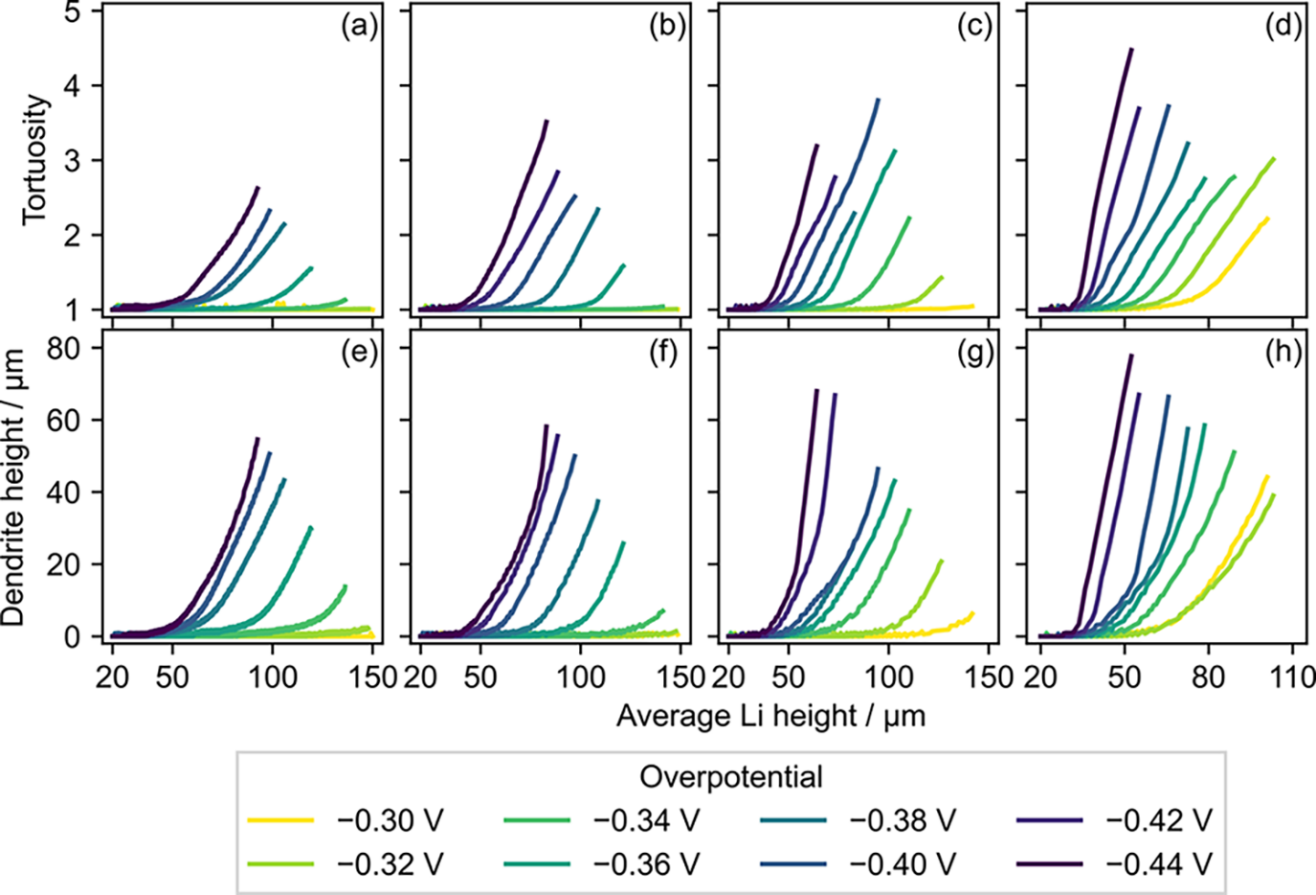
Evolution of (a–d) tortuosity and (e, f) dendrite height as a function of average Li height at (a, e) 273 K, (b, f) 293 K, (c, g) 313 K, and (d, h) 333 K. The tortuosity and dendrite height are shown until the peak Li height reaches 150 μm.

The discussion in this section is limited to the case where the average Li height is 55 μm for a systematic assessment of the surface modulation. While the comparison is made on the same average Li height, as the same height corresponds to the same amount of Li deposited on the electrode, the scope is limited to a single Li height. The evolution of tortuosity and dendrite height as a function of average Li height is shown in Figure 7, where the curves are terminated when the Li peak reaches a height of 150 μm. It can be seen that the average Li height at the onset of the dendrite formation (in terms of both tortuosity and dendrite height) becomes lower as the magnitude of the overpotential and temperature are increased, agreeing with the patterns observed so far. Additionally, both the tortuosity and dendrite height increase almost linearly with respect to the average Li height beyond the onset point, and their slopes are quite similar across the entire range of overpotential and temperature. The linear increase of tortuosity and dendrite height indicates that it is critical to compare the onset point of the dendrite formation, since they continue to grow at a rather predictable rate afterward.

### 3.3 Influence of Temperature and Overpotential on the Onset Point of Dendrite Formation

**Figure 8:**
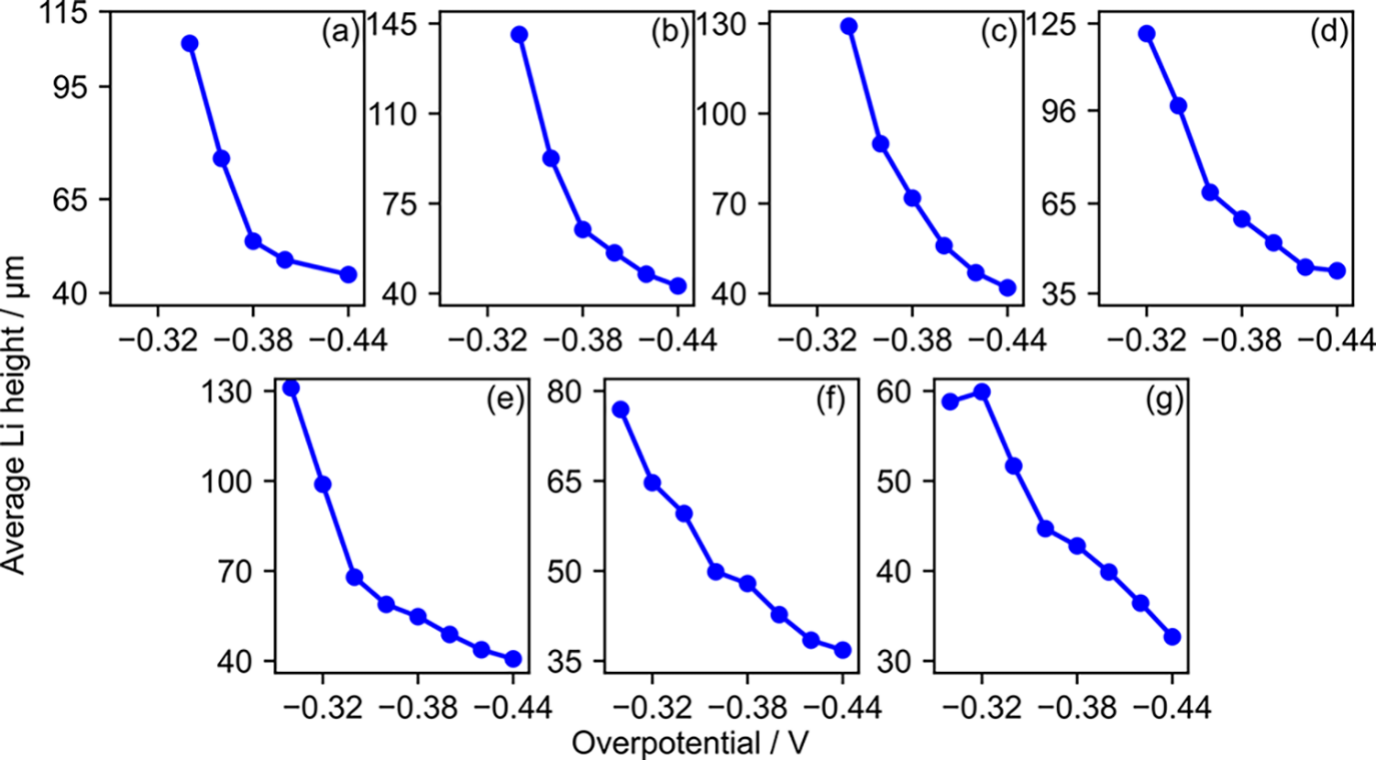
Average height of an Li electrode at the onset of dendrite formation when the temperature is (a) 273 K, (b) 283 K, (c) 293 K, (d) 303 K, (e) 313 K, (f) 323 K, and (g) 333 K.

We have established threshold criteria on tortuosity and dendrite height to determine the onset point at which dendrites start to form and demonstrated that it is critical to determine the onset point. It is shown in Figure 8 that the onset of the dendrite formation decreases almost monotonically as the magnitude of the applied overpotential is increased for all temperatures. The pattern makes intuitive sense, as applying a higher overpotential to drive the electrodeposition is more likely to introduce an inhomogeneity on the surface while it allows less time for the surface ions to diffuse along the surface. Therefore, it is desirable to reduce the magnitude of the overpotential to suppress dendrite formation. The conclusion drawn our simulation results agrees well with the existing experimental reports where the increased current density promotes dendrite formation.^15,16^ As shown in section 3.2, the higher overpotential translates to higher current density, allowing one to make a qualitative comparison between potentiostatic and galvanostatic measurements to assess the influence of overpotential/current density on dendrite growth.

**Figure 9:**
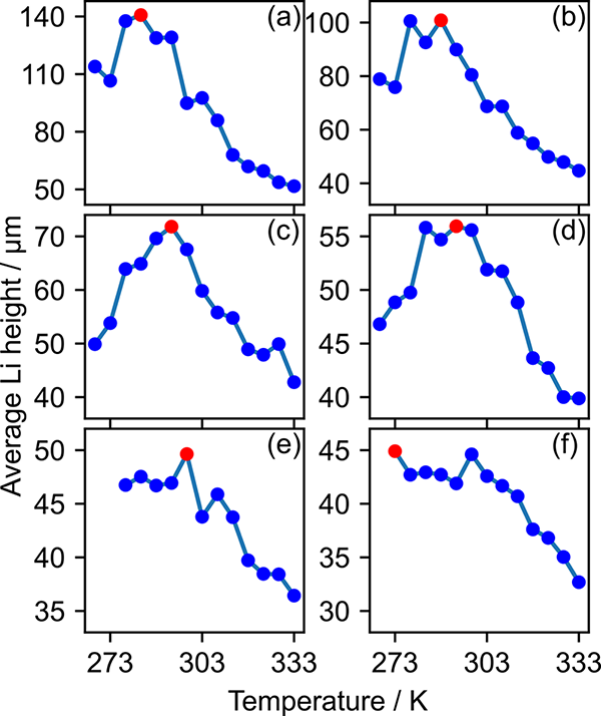
Average height of the Li electrode at the onset of dendrite formation when the applied overpotential is (a) −0.34 V, (b) −0.36 V, (c) −0.38 V, (d) −0.40 V, (e) −0.42 V, and (f) −0.44 V. Red points indicate the temperatusre at which the average Li height reaches its maximum value.

The temperature dependence of the onset point, on the other hand, is not as straightforward as that of the overpotential. As shown in Figure 9, there is no monotonic increase or decrease in the onset point as a function of temperature. Note that the temperature dependence of the onset point is not reported for ϕ = −0.30 and −0.32 V, as dendrites do not form at low temperatures. Despite some fluctuations in the pattern, it can be seen in Figure 9 that the onset point increases as the temperature increases in the low-temperature region and decreases in the high-temperature region. The temperature at which the onset point reaches its peak height is the optimal operating temperature that inhibits the formation of dendrites. The optimal temperature gradually shifts from 283 to 298 K as the applied overpotential is increased from −0.34 to −0.42 V. A slight anomaly of a sudden decrease in the optimal temperature to 273 K is observed when the overpotential is −0.44 V (Figure 9f). However, the difference between the highest average height at 273 K and the second-highest average height at 293 K is sufficiently low to consider them to be within the error margin. The presence of the optimal temperature between 283 and 298 K agrees well with the temperature dependence of tortuosity and dendrite height discussed in section 3.1, where the temperature increase at low temperatures resulted in a decrease in dendrite height and reached its minimum at temperatures between 278 and 298 K, indicating the nonlinear dependence of temperature for the transition from a reaction-limited to a transport-limited process. A similar nonlinear dependence on temperature is observed for onset points, since it is determined using dendrite height and tortuosity thresholds.

The optimal operating conditions do not exceed room temperature regardless of the applied overpotential value. A further increase in temperature lowers the onset point of the dendrite formation, which implies that increasing the temperature beyond room temperature promotes the dendrite formation. Such a pattern is in contrast with previous reports where elevating the temperature is found to be beneficial for dendrite suppression.^26,31^ As pointed out previously, the difference stems from the fact that these studies are carried out under galvanostatic conditions where the increase in temperature lowers the overpotential across the interface. In contrast, potentiostatic simulations are carried out in this work. The elevated temperature causes the induced current to increase, making it difficult to compare the dendrite suppression observed in galvanostatic measurements directly. Such a discrepancy highlights that care must be taken when the thermal effects observed under galvanostatic and potentiostatic conditions are compared.

## 4 Conclusions

A nonlinear phase-field model that integrates thermal effects is presented. The thermal effect was reflected via the use of temperature-dependent values of the electrode and electrolyte conductivity, surface tension, exchange current density, and Li-ion diffusivity in the electrolyte. The correlation between the Li-ion concentration profile at the interface and the dendrite formation is verified, and the correlation is used to set thresholds on surface tortuosity and dendrite height that defines the onset condition for dendrite formation. It was found that elevating the temperature increases the induced current and accelerates the electrodeposition process. The increase in temperature and overpotential promotes dendrite formation. The onset point for the dendrite formation is a crucial factor to consider, since the rate of dendrite growth with respect to the amount of deposited Li is similar across all temperatures and overpotentials. It was determined that increasing the overpotential lowers the onset point. The temperature dependence of the onset point was not linear, and the optimal operating temperature was observed to increase as the magnitude of the overpotential was increased. A deeper insight into the underlying mechanism of dendrite formation and growth can be gained in the future via a combined potentiostatic and galvanostatic investigation using a phase-field model that incorporates the internal heat generation, convection, and radiation.
